# BiG-FAM: the biosynthetic gene cluster families database

**DOI:** 10.1093/nar/gkaa812

**Published:** 2020-10-03

**Authors:** Satria A Kautsar, Kai Blin, Simon Shaw, Tilmann Weber, Marnix H Medema

**Affiliations:** Bioinformatics Group, Wageningen University, 6708PB Wageningen, The Netherlands; The Novo Nordisk Foundation Center for Biosustainability, Technical University of Denmark, 2800 Kgs. Lyngby, Denmark; The Novo Nordisk Foundation Center for Biosustainability, Technical University of Denmark, 2800 Kgs. Lyngby, Denmark; The Novo Nordisk Foundation Center for Biosustainability, Technical University of Denmark, 2800 Kgs. Lyngby, Denmark; Bioinformatics Group, Wageningen University, 6708PB Wageningen, The Netherlands

## Abstract

Computational analysis of biosynthetic gene clusters (BGCs) has revolutionized natural product discovery by enabling the rapid investigation of secondary metabolic potential within microbial genome sequences. Grouping homologous BGCs into Gene Cluster Families (GCFs) facilitates mapping their architectural and taxonomic diversity and provides insights into the novelty of putative BGCs, through dereplication with BGCs of known function. While multiple databases exist for exploring BGCs from publicly available data, no public resources exist that focus on GCF relationships. Here, we present BiG-FAM, a database of 29,955 GCFs capturing the global diversity of 1,225,071 BGCs predicted from 209,206 publicly available microbial genomes and metagenome-assembled genomes (MAGs). The database offers rich functionalities, such as multi-criterion GCF searches, direct links to BGC databases such as antiSMASH-DB, and rapid GCF annotation of user-supplied BGCs from antiSMASH results. BiG-FAM can be accessed online at https://bigfam.bioinformatics.nl.

## Graphical Abstract

**Graphical Abstract:**
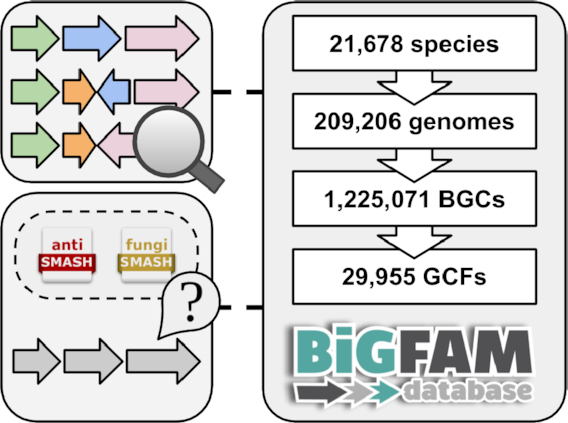
Constructed based on a large-scale homology analysis of 1.2 million biosynthetic gene clusters, BiG-FAM provides a platform to explore their genomic diversity and to discover their relationships to newly sequenced ones.

## INTRODUCTION

Microbial secondary metabolism produces a vast array of natural products (NPs) beneficial not only to the microbes themselves, but sometimes also to humans, for use as, e.g. antibiotics, chemotherapeutics, and crop protecting agents (1,2). Enzyme-coding genes for these metabolic pathways, as well as genes encoding associated transporters and regulators, are often found physically co-located within a microbial genome, on loci referred to as biosynthetic gene clusters (BGCs). With the increasing availability of bacterial and fungal genome sequences, BGC identification tools like antiSMASH (3) and PRISM (4) have played a critical role in transforming NP discovery into a genome-based endeavor, as they allow the investigation of bioactive compounds a microorganism may produce even if the pathways are not expressed in the lab or when the genomes originate from uncultivated organisms.

With the simultaneous sequencing of hundreds to thousands of microbial genomes becoming more common, the large quantities of BGC data resulting from this pose an opportunity as well as a challenge. Databases such as antiSMASH-DB (https://antismash-db.secondarymetabolites.org/) (5), IMG-ABC (https://img.jgi.doe.gov/cgi-bin/abc/main.cgi) (6) and MIBiG (https://mibig.secondarymetabolites.org/) (7) have a crucial role in the analysis of BGCs, as they allow comparing the sequences of newly sequenced BGCs against those of previously predicted and experimentally characterized ones (8). However, while this sequence-based approach works well to identify closely related BGCs across different taxa (9,10), it does not facilitate global analysis of relationships between BGCs across taxa. This is exemplified by the ClusterBlast module in antiSMASH (3), which, for every detected BGC, outputs a visualized overview of an arbitrary number of top hits for its sequence similarity searches, while the actual number of homologous gene clusters may be smaller or (much) larger.

To identify groups of BGCs that are functionally closely related and encode the production of the same or very similar molecules, approaches have been developed to group BGCs into gene cluster families (GCFs) (10–12). GCFs have been shown to be very useful in genome-based NP discovery efforts. By examining shared absence/presence patterns of GCFs and compound families (derived via molecular networking of MS/MS spectra (13–15)) across different microbial strains, one can connect BGCs to their expressed products (12,13,16–19). Algorithms such as BiG-SCAPE (19) automate the GCF reconstruction process and provide detailed interactive sequence similarity networks that can be explored by the users. However, sequence similarity network approaches are not sufficiently scalable to perform global analysis of all BGCs publicly available data. Recently, we developed BiG-SLiCE (20), a tool that makes it possible to perform GCF reconstruction at very large scales in a computationally feasible way. While BiG-SCAPE uses a pairwise comparison strategy to build GCF networks of up to ∼70,000 BGCs within 10 days in a compute server, BiG-SLiCE applies BGC vectorization coupled with a near-linear clustering algorithm to process more than a million BGCs under the same computational runtime.

Here, we present BiG-FAM, an online database that leverages BiG-SLICE clustering of BGCs to enable GCF-based exploration and homology searching of >1.2 million BGCs harbored by >200,000 microbial genomes. Thus, it provides a complete picture of the ‘global’ secondary metabolic diversity of microbial NPs. Using this web-based platform, scientists can explore the biosynthetic repertoires of specific taxa, investigate the taxonomic and architectural diversity of BGCs of known function, and obtain insights into the novelty of newly sequenced BGCs or their relationships to BGCs in publicly available genomes. For further analysis of the underlying BGCs, BiG-FAM provides cross-links to both the MIBiG and antiSMASH databases, which contain more detailed annotations on the BGCs, such as their predicted or characterized chemical products.

## DATABASE FEATURES

### GCF data on over 1.2 million BGCs

**Figure 1.**
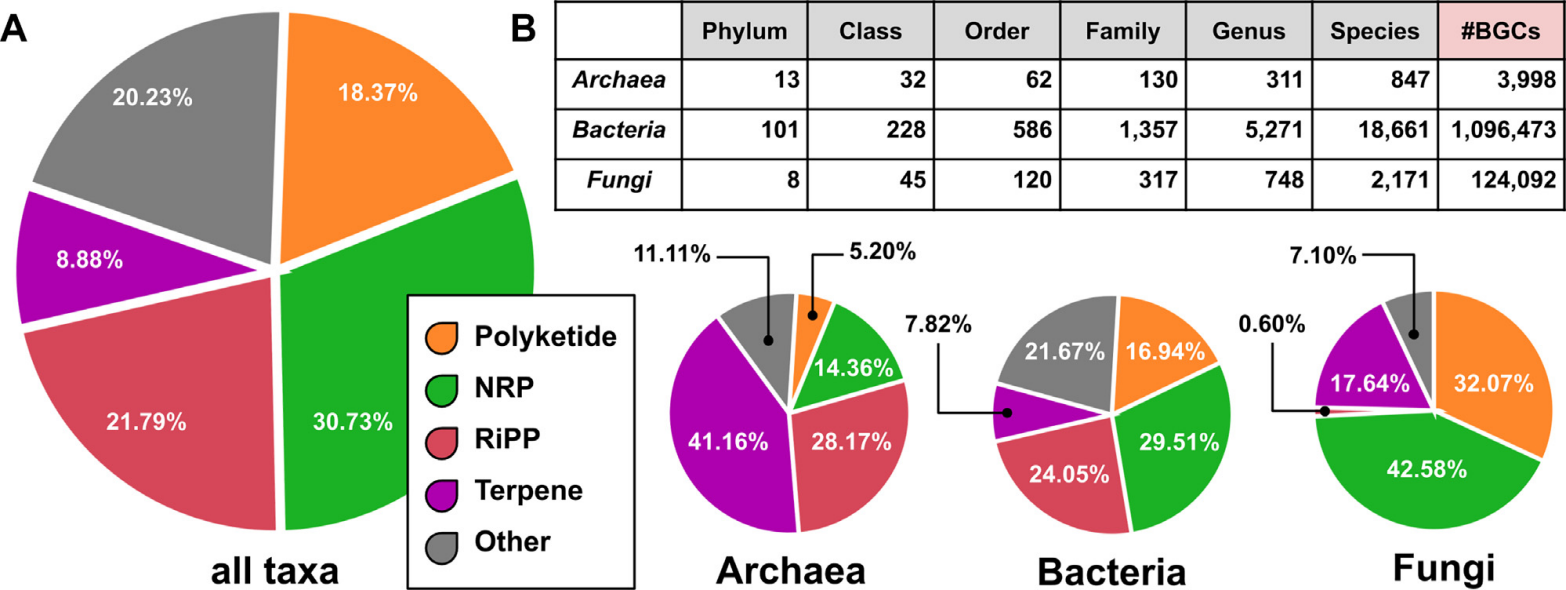
(**A**) Pie chart depicting the ratio of five generic BGC classes within the full dataset across three different microbial kingdoms (a total of 1,224,563 BGCs, excluding 16 plant BGCs from MIBiG and 492 BGCs with unassigned taxonomy). (**B**) Taxa covered by BGCs in BiG-FAM (total number of unique taxa represented by at least one BGC-containing genome per taxonomy level), with the total number of BGCs per kingdom provided in the far-right column of the table.

The BiG-FAM database contains 29,955 GCFs previously calculated using BiG-SLiCE version 1.0 (parameters ‘–complete-only –threshold 900’) from a collection of 1,225,071 BGCs (20). These ∼1.2 million BGCs were predicted by antiSMASH v5.1.1 from a set of 188,622 microbial genomes (181,521 bacterial, 5,939 fungal and 1,162 archaeal genomes from the NCBI RefSeq/GenBank database) and 20,584 MAGs (from several previously published studies (21–25)), and then complemented with 1,910 experimentally characterized BGCs from the MIBiG 2.0 database (7). A complete list of all genomes along with their BGC counts and taxonomy according to the Genome Taxonomy Database (GTDB) (26) is provided in Supplementary Table S1. The included BGCs (and their corresponding GCFs) cover a wide range of biosynthetic classes (Figure 1A) and taxa (Figure 1B), thus providing extensive coverage of the secondary metabolic diversity of the ‘observable microbial universe’.

### Seamless exploration of the database content

**Figure 2.**
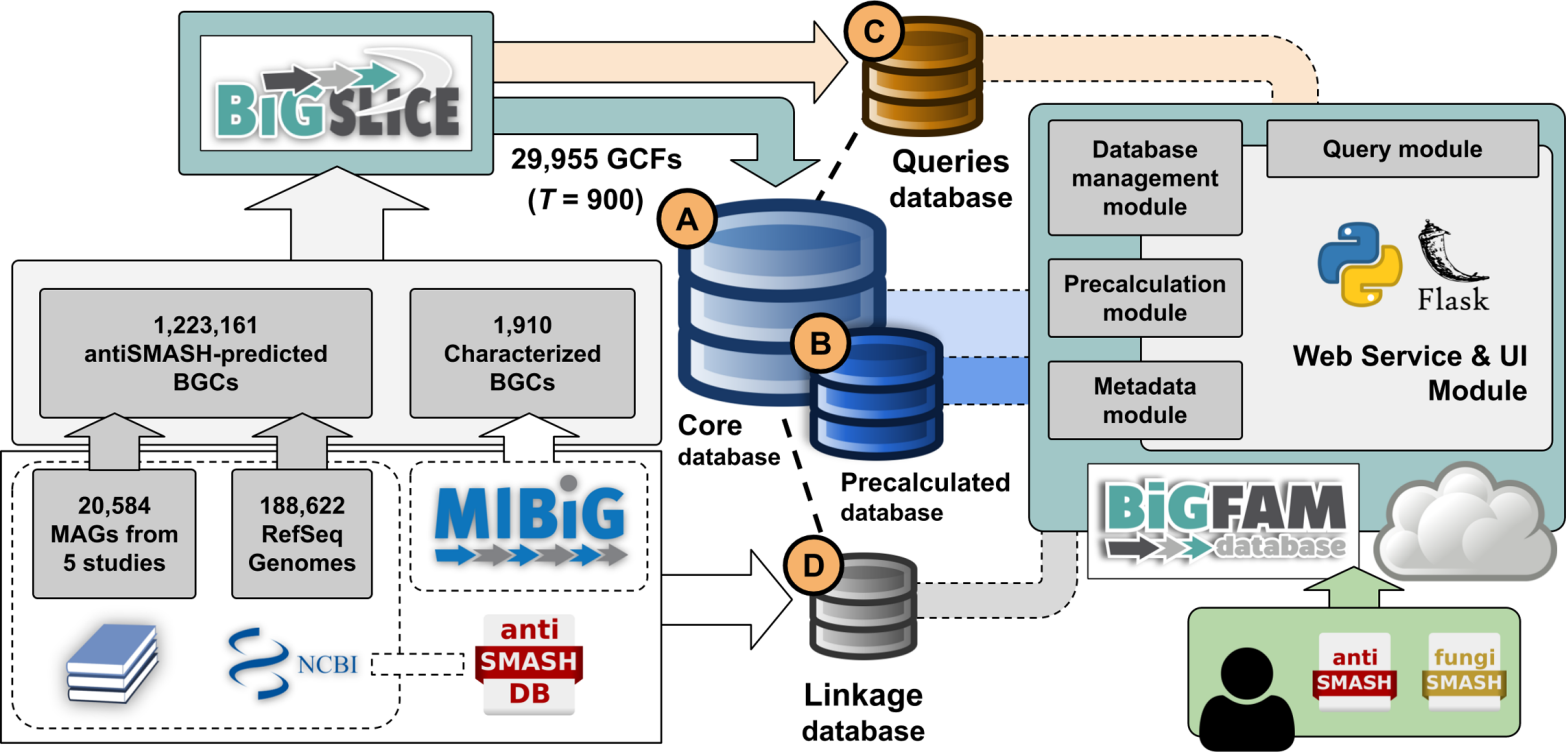
Workflow schema of BiG-FAM’s architecture. Starting from a collection of ∼1.2 million BGCs, BiG-SLiCE was used to perform a clustering analysis (with threshold parameter *T* = 900), resulting in (**A**) 29,955 GCFs stored in an SQLite3 database file. This file is used as the ‘Core database’ for BiG-FAM. To support BiG-FAM’s extensive functionalities, three related database files were created, each managed by a specific module in the software package. (**B**) The ‘Precalculated database’ summarizes complex SQL operations (i.e., calculation of taxonomy counts per GCF) to speed up page loads (detailed schema and procedures can be accessed from the ‘precalculation’ module in BiG-FAM’s source code). (**C**) The ‘Queries database’ stores information related to user-submitted BGC queries, such as processed features from antiSMASH BGCs and the corresponding list of best-matched GCFs identified using BiG-SLiCE. (**D**) Finally, the ‘Linkage database’ keeps tab on the cross-links to external databases (i.e. MIBiG and antiSMASH-DB), storing information such as the accession number of each linked BGC, which can be used to generate the correct URL addresses pointing to the correct entry within the specific database. These modules and databases were used to serve an online database written in Python using the Flask programming library.

Starting with the core SQLite3-based (https://sqlite.org/index.html) data storage produced by BiG-SLiCE version 1.0.0 (Figure 2A), we built a fully functional web server using the Python Flask library (https://palletsprojects.com/p/flask/). We implemented an extra layer of cache storage (Figure 2B) to prefetch complex SQL queries coming from various parts of the web server. This, in turn, provides a seamless browsing experience at ‘compute-heavy’ web pages, such as the (database-wide and per-GCF) ‘Statistics’ view. Furthermore, this setup allowed a fairly lightweight (each request is returned within 0.5–5 s on average) implementation of the ‘Search and Filter’ function on both BGCs and GCFs, giving users the ability to look for BGCs or GCFs annotated with specific taxa, source dataset types, biosynthetic classes or protein domains.

### Querying user-supplied BGCs for rapid GCF placement

One major advantage of using BiG-SLiCE is that the shared BGC features of each GCF are summarized in the same Euclidean-based feature matrix as used for the underlying BGCs, forming what are known as the GCF models (or GCF centroids). This in turn enabled a linear BGC-to-GCF matching, which allows placing dozens of newly sequenced BGCs from a typical microbial genome onto the global map of precalculated biosynthetic diversity within seconds of compute time. To enable easy access to this powerful feature, BiG-FAM incorporates a web-based ‘Query’ submission system, for which the supporting data infrastructure is stored in a separate database (Figure 2C), where users can directly take their antiSMASH-predicted BGCs (using job IDs from the antiSMASH web server) and submit them for a GCF analysis. The produced BGC-to-GCF hits will reveal the close (i.e. near-duplicate) and distant relatives of the queried BGCs, which provides insights into their novelty and their relationships to other BGCs and helps studying their distribution and evolution across taxa.

### Direct links to BGC and genome databases

While BiG-FAM stores and displays a lot of useful BGC-related information (such as protein sequences and biosynthetic domain hits), the database was never intended to be a BGC database, and therefore does not include information not directly related to the GCFs, such as nucleotide sequences of the BGCs. To support users who are looking for these data, BiG-FAM stores links to 1,910 known BGCs from MIBiG and 43,117 NCBI-derived BGCs from the antiSMASH database as metadata (Figure 2D, Supplementary Table S2). These cross-links can be used to acquire, for example, further information about the characterized (i.e., user-curated information from the MIBiG database) and predicted (for BGCs in antiSMASH database) core structure of the BGC products. Finally, links to the original genome sources (NCBI nucleotide database for isolate datasets, publication's URL for MAG datasets) are also accessible from each BGC’s summary page (or as a merged URL list available for download from the GCF page).

## EXAMPLE USE CASES

There are several ways in which BiG-FAM can be used to answer scientific questions related to microbial secondary metabolism. The ‘Search and Filter’ function can be used to track the distribution of specific groups of BGCs (i.e. based on their generic classes, or the queried combination of their biosynthetic domains) across different taxa. Alternatively, the ‘Query’ page can be used to rapidly match user-supplied BGCs against the set of precalculated GCFs, providing useful information for the characterization and dereplication of those BGCs. Here, we describe two real-world use cases to demonstrate how such analyses could be done by the database users.

### Example use case 1: exploring ranthipeptide BGC diversity

**Figure 3.**
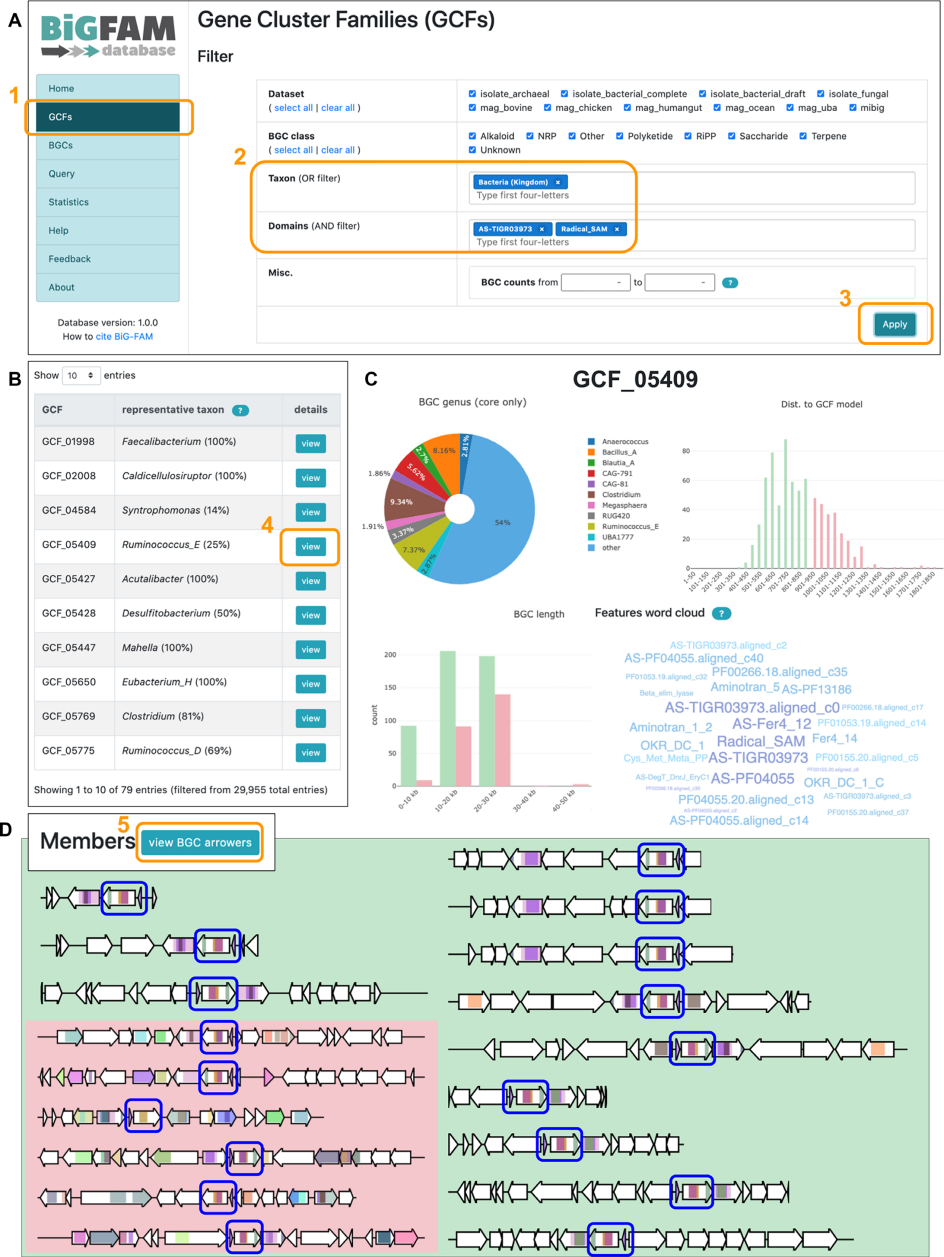
(**A**) By clicking on the ‘GCF’ page link (box 1) from the main menu, users will be provided with an interface to search GCFs based on multiple criteria; in this case we search for ‘bacterial GCFs harboring AS-TIGR03973 and Radical_SAM biosynthetic domains in at least ∼80% of their BGCs’ (box 2). (**B**) After applying the filter function (box 3), BiG-FAM returned a list of 79 GCFs satisfying the criteria. (**C**) Clicking on the ‘view’ button of a GCF (box 4) will take users to a detail page that shows several statistics related to the GCF’s taxonomic distribution, length of its BGCs, and features (domains) distribution. (**D**) In the GCF detail page, users may also choose to view an ‘arrower’ visualization of the BGCs (box 5), which in this case shows the occurrence of neighboring biosynthetic genes (depicted in colored arrows) flanking the queried cysteine-rich precursor + rSAM gene pairs (blue boxes).

Ranthipeptides (previously known as ‘SCIFF peptides’) are ribosomally synthesized and post-translationally modified peptides (RiPPs) prevalent in the taxonomic class *Clostridia* (27), although GC content analysis indicated that their biosynthetic genes might be horizontally transferred to other taxa as well (28). Recent analysis shows that these peptides played an important role in regulation at the population level, i.e. via a quorum sensing mechanism (29). During our previous effort in charting the global diversity of 1.2 million BGCs (20), we captured a large group (6,800) of putative ranthipeptide BGCs with diverse patterns of gene neighborhoods flanking the precursor peptides. To explore this diversity, we can use BiG-FAM’s ‘GCF search’ function and use the two signature domains of this BGC class (AS-TIGR03973 and Radical_SAM) as query baits (Figure 3A). The search result shows 79 GCFs, each representing a distinct pattern of underlying BGCs distribution across taxonomy (Figure 3B). By clicking on the link to each GCF’s detail page, information is provided about the taxonomic origins, nucleotide length, calculated radius and biosynthetic features shared by BGCs within the GCF (Figure 3C). Additionally, an overview of all BGCs and links to obtain their sequences can be downloaded in TSV format. Furthermore, a comparative multi-gene visualization of the BGCs provides a collated view on the diversity of gene neighborhoods flanking the ranthipeptide precursor genes (Figure 3D).

### Example use case 2: GCF analysis on a newly sequenced *Streptomyces* strain

**Figure 4.**
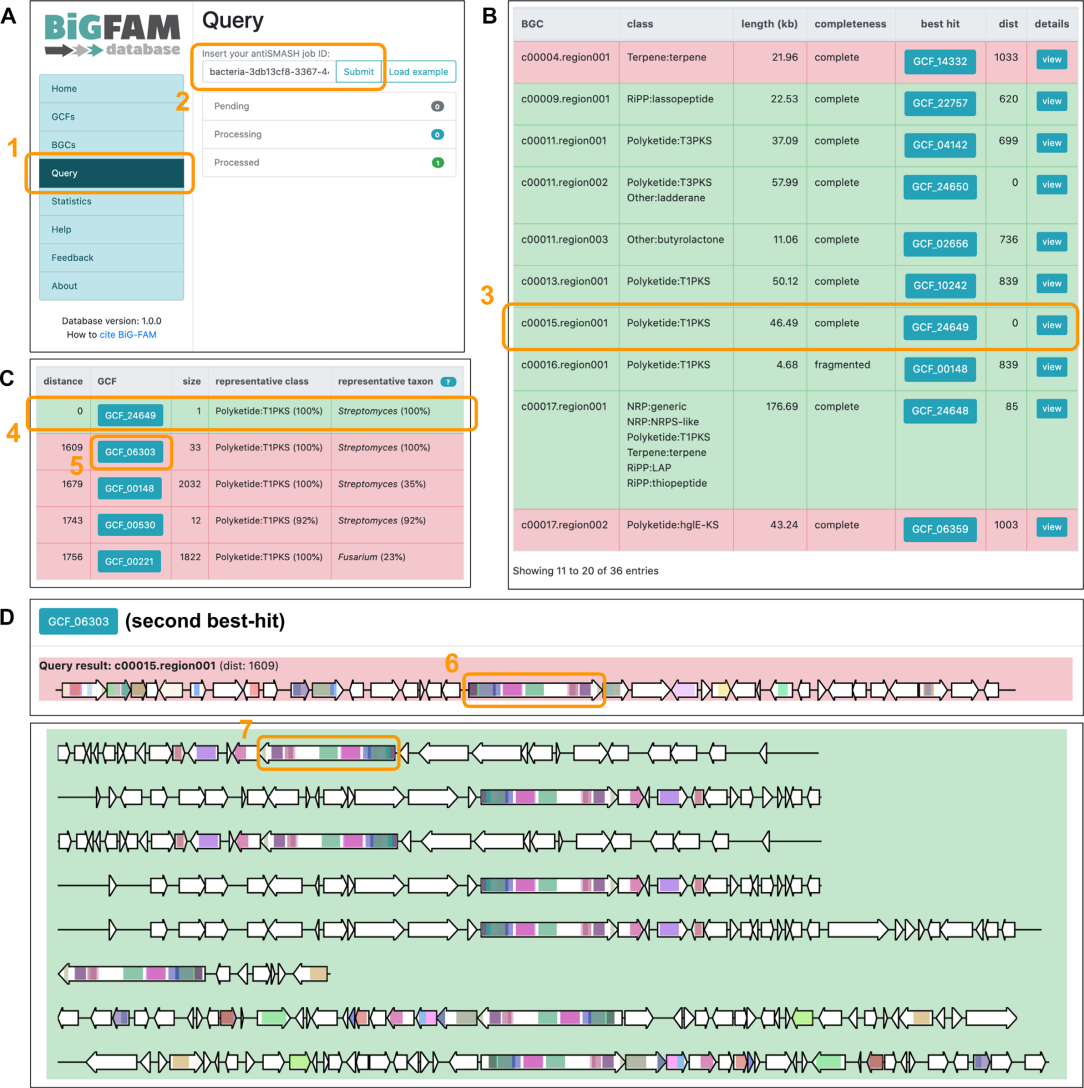
(**A**) When users click on the ‘Query’ section of the main menu (box 1), they will be presented with a form to input the job ID of a finished antiSMASH run. After pressing ‘Submit’, BiG-FAM will immediately execute (or put into queue) the downloading, preprocessing and GCF matching of all BGCs (i.e. regions) included in the submitted run. (**B**) A list will then be shown with the summary of all best BGC-to-GCF pairings with distance lower than 900 (original threshold value) highlighted in green, depicting a good match to at least one GCF in the database. A particular query BGC, ‘Region 15.1’ was selected for a detailed look (box 3) as mentioned in the main text. (**C**) A list of five best-matching GCFs and their model distances to the query BGC, showing an exact match (*d* = 0) to a singleton GCF from *Streptomyces* (GCF_24649, box 4) which turned out to be the same BGC from the same genome. Looking at the visualization of the second closest GCF on the list (GCF_06303 with *d* = 1609, box 5), we can see (**D**) co-occurrence of protein domains across the distantly related BGCs, where some similar but non-identical PKS genes (longest multi-domain gene in each GCF) seems to act as an ‘anchor’ that defines the GCF. While this group of anchor genes have a similar domain architecture to the PKS gene of the queried BGC (box 6), a quick BLASTp analysis against one example gene (box 7) shows only 52.63% amino acid identity (Supplementary Text 1). Along with the differences in non-PKS genes between the query BGC and the gene clusters in the GCF, this suggests that, while the BGC is (distantly) related to this GCF, it does not actually belong to it and constitutes a novel gene cluster architecture.

Recently, a draft genome was published (30) for *Streptomyces tunisialbus*, a new streptomycete isolated from the rhizospheric soil of lavender plants (*Lavandula officinalis*) in Tunisia (31). To showcase how BiG-FAM can be used to assess biosynthetic novelty and capture distant relationships of newly sequenced BGCs, we downloaded the assembled genome from ENA (accession: OKRJ01) and uploaded it to the antiSMASH web server (http://antismash.secondarymetabolites.org/), returning a unique job id (‘bacteria/fungi-xxxxxxxx-xxxx-xxxx-xxxx-xxxxxxxxxxxx’) which (after the run is done) can then directly be used to perform GCF analysis in BiG-FAM (https://bigfam.bioinformatics.nl/query) (Figure 4A). The entire analysis for the 36 antiSMASH-predicted BGCs was completed in less than a minute, resulting in a summary table of the best BGC-to-GCF hit pairs (Figure 4B). One interesting BGC in this genome is the complete, 46.5 kb long Type-I PKS protocluster from ‘Region 15.1’, which shows an overall low hit rate to gene clusters from public databases in both its ClusterBlast and KnownClusterBlast results (Supplementary Figure S1). A quick look at the GCF analysis result for the BGC shows a significant hit only to one singleton GCF (Figure 4C), which after a follow-up inspection turned out to originate from the NCBI-submitted entry of the same genome (accession: GCA_900290435.1). This suggests that the PKS BGC in question represents a novel type of BGC, as it is not closely related to any GCFs with members from other genomes. Relationships to more distantly related GCFs and BGCs can be analyzed by ‘tracking’ of biosynthetic domains of the query BGC across hundreds to thousands of distant BGCs, showing the domain architectural similarity shared between the genes (Figure 4D).

## DISCUSSION

Being the first resource to offer unprecedented access to the ‘global’ biosynthetic space of microbial BGC families, we expect BiG-FAM to become a relevant resource for NP discovery. With its feature-rich web interface, BiG-FAM facilitates user-friendly exploration and querying of its GCFs and BGCs. In the future, ‘Wikipedia-style’ manually curated or semi-automatically generated annotations of precalculated GCFs (e.g. based on the presence of known BGCs or enrichment of signature proteins) may make the database even more useful for end-users. Moreover, with the recent emergence of metadata-rich microbial NP structure databases like the NPAtlas (https://www.npatlas.org/) (32) and databases of mass-spectrometric data like GNPS (33), it may become feasible to perform global meta-analyses to link (taxonomically conserved) BGCs to compounds based on their species/genus level presence/absence patterns observed across these databases (34).

To improve BiG-FAM in subsequent releases, additional useful features for users are planned, such as a REST-based API to support programmatic access to the data and more detailed downloadable summaries (e.g. in a tab-separated text file) that can be used for downstream analyses of the GCFs. Furthermore, there are opportunities to further extend the coverage of precalculated BGCs and GCFs in BiG-FAM by incorporating additional data sources. For example, IMG-ABC currently holds >400,000 BGCs from their >60,000 bacterial genomes, with some degree of overlap against NCBI and MIBiG. There are also other microbial genome databases like MycoCosm (35) that contain genomes not submitted to GenBank or the ENA. Finally, future efforts should also incorporate more data from shotgun metagenomic studies to cover a greater extent of the unculturable microbial biosphere.

## DATA AVAILABILITY

The BiG-FAM database is publicly available online and can be accessed without login requirements at https://bigfam.bioinformatics.nl, while the Python script and SQLite schema used to construct the database is available as open source at https://github.com/medema-group/bigfamdb. All data in BiG-FAM are freely available under the Creative Commons CC-BY license.

## ABBREVIATIONS

**NP:** Natural Product. **BGC:** Biosynthetic Gene Cluster. **GCF**: Gene Cluster Family. **MAG**: Metagenome-Assembled Genome. **AntiSMASH-DB**: AntiSMASH Database (https://antismash-db.secondarymetabolites.org/). **IMG-ABC**: Integrated Microbial Genomes – Atlas of Biosynthetic Gene Clusters (https://img.jgi.doe.gov/cgi-bin/abc/main.cgi). **MIBiG**: Minimum Information about a Biosynthetic Gene Cluster (https://mibig.secondarymetabolites.org/). **GTDB**: Genome Taxonomy Database (https://gtdb.ecogenomic.org/). **URL:** Uniform Resource Locator (i.e. a website address). **NCBI**: National Center for Biotechnology Information (https://www.ncbi.nlm.nih.gov). **SCIFF**: Six-Cysteine in Forty-Five (peptide). **rSAM:** radical *S*-adenosylmethionine. **RiPP**: Ribosomally synthesized, Post-translationally modified Peptide. **ENA**: European Nucleotide Archive (https://www.ebi.ac.uk/ena). **PKS**: Polyketide Synthase. **REST**: Representational State Transfer (software architecture). **API**: Application Programming Interface.

## Supplementary Material

gkaa812_Supplemental_Files
