# Supplementary material for: BiG-FAM: the biosynthetic gene cluster families database
Source: Nucleic Acids Res. 2020 Oct 3;49(D1):D490–7. doi: 10.1093/nar/gkaa812 (PMC7778980; doi:10.1093/nar/gkaa812)
Supplement: gkaa812_Supplemental_Files [file gkaa812_supplemental_files.zip › supplementaries_captions.docx]

**Supplementary Table 1.** List of all genomes in BiG-FAM along with their BGC counts and GTDB-assigned taxonomy information.

**Supplementary Table 2.** List of BGCs in BiG-FAM with corresponding antiSMASH-DB metadata.

**Supplementary Text 1.** Blastp analysis result between PKS gene of the queried BGC (Region 15.1) against another PKS gene in GCF_06303 (a Type-I PKS BGC from *Streptomyes rimosus*).

**Supplementary Figure 1.** **A.** ClusterBlast and **B.** KnownClusterBlast results of the Region15.1 BGC, showing low overall hits (4% - 30% MultiGeneBlast scores) from both databases. Notably, four PKS and geranylgeranyl reductase (GGR) pairs from multiple *Streptomyces* species (blue boxes) do show some similarity (~80% protein similarity for each gene) to the PKS+GGR of the query BGC (red box).
