# Supplementary figures and images for: BiG-FAM: the biosynthetic gene cluster families database
Source: Nucleic Acids Res. 2020 Oct 3;49(D1):D490–7. doi: 10.1093/nar/gkaa812 (PMC7778980; doi:10.1093/nar/gkaa812)

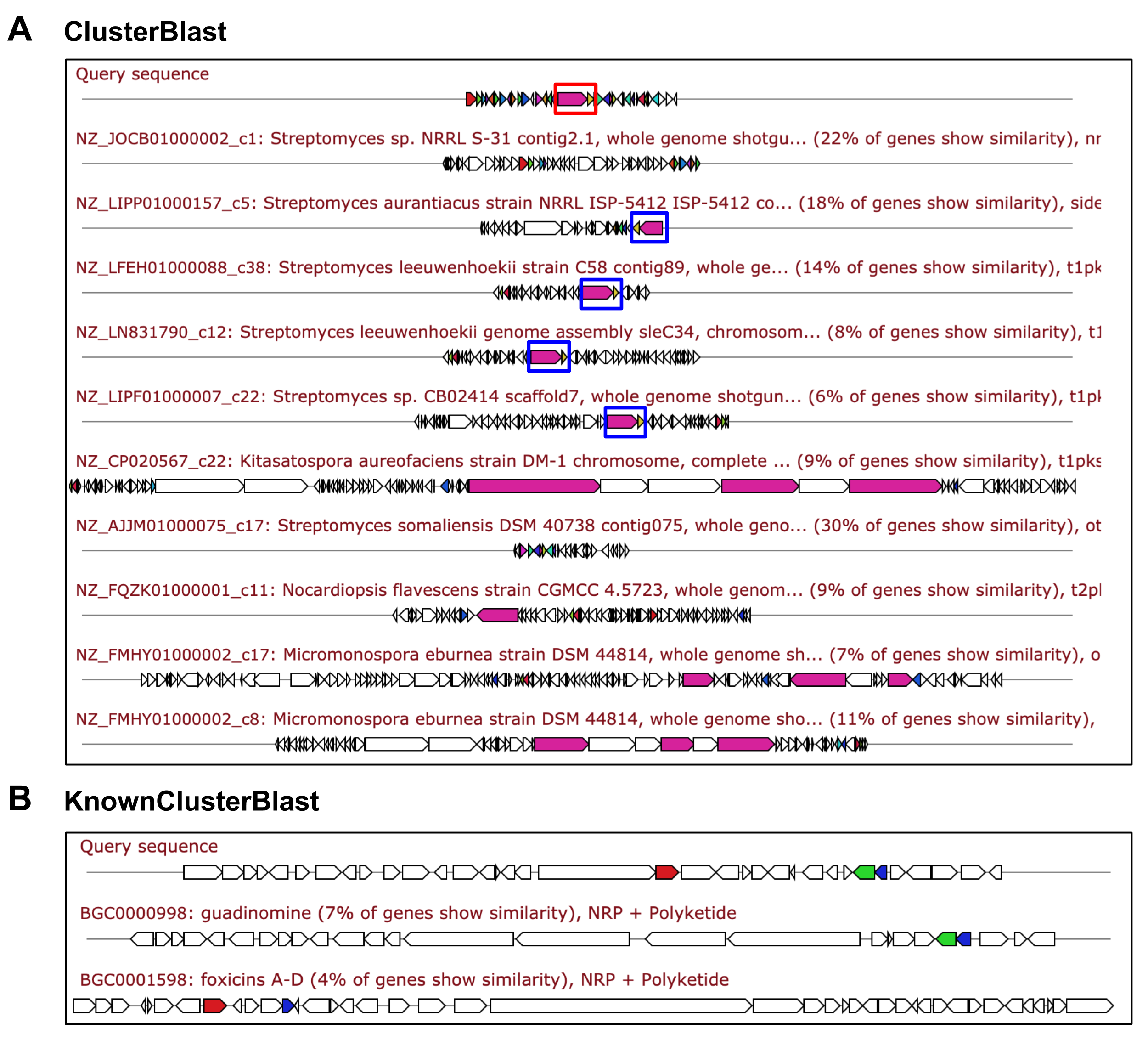

Supplement: gkaa812_Supplemental_Files [file gkaa812_supplemental_files.zip › supplementary_figure_1.png]
